# Supplementary material for: Assessment of the Genetic Characteristics of a Generation Born during a Long-Term Socioeconomic Crisis
Source: Genes (Basel). 2023 Nov 11;14(11):2064. doi: 10.3390/genes14112064 (PMC10671057; doi:10.3390/genes14112064)
Supplement: Supplementary file 1 [file genes-14-02064-s001.zip › genes-2686799-supplementary.pdf]

**Table S1.** Conditions for PCR, R: the number of repeats in a VNTR

| Gene, polymorphism | PCR primers | Annealing temperature, °C | Amplicons' lengths, bp |
|---|---|---|---|
| *MAOA*, VNTR in promoter | 5′-ACAGCCTGACCGTGGAGAAG-3′<br>5′-GAACGGACGCTCCATTCGGA-3′ | 63 | 290 (2R), 320 (3R), 335 (3.5R), 350 (4R), 380 (5R) |
| *SLC6A3*, VNTR in 3′-UTR | 5′-TGCGGTGTAGGGAACGGCCTGA-3′<br>5′-CTTCCTGGAGGTCACGGCTCAAGG-3′ | 64 | 403 (8R), 443 (9R), 483 (10R), 523 (11R) |

**Table S2.** Oligonucleotide and TaqMan probe sequences used for real-time PCR analysis of rs17689918, rs1360780, rs53576, rs12720071, rs806377, rs6557168, rs4311, rs4522666, and rs1800497

| SNV | Oligonucleotide and TaqMan probe sequences | $T_m$, °C |
|---|---|---|
| rs17689918 | 5′-GGTTGTTCAGGCTGTGACTGG-3′<br>5′-CACCACACCTGTCACCCAGT-3′<br>[FAM]CAGATTCAGGCTGAGATTGCGAAC[BHQ1]<br>[HEX]CAGATTCAGGCTGAGATTGCAAA[BHQ1] | 60 |
| rs4311 | 5′-AAGTCTTCCCCAGTTCCTCA-3′<br>5′-GATGGTTAAATTGTCTTAGGGCC-3′<br>[FAM]AGAGGTTGCAAACAGATGGATTTAACT[BHQ1]<br>[HEX]AGAGGTTGCAAACAGATGGATTTAGCT[BHQ1] | 59 |
| rs1360780 | 5′- CAGAAGAGATCCAGGCACAG-3′<br>5′- GCCAGCAGTAGCAAGTAAGA-3′<br>[FAM]AGGCTTTCACATAAGCAAAGTTATAC[BHQ1]<br>[HEX]AGGCTTTCACATAAGCAAAGTTACAC[BHQ1] | 60 |
| rs1800497 | 5′-GCAACACAGCCATCCTCAAAGTG-3′<br>5′- CAGCTCACTCCATCCTGGACGT-3′<br>[FAM]CAGCTGGGCGCCTGCCTCGACC[BHQ1]<br>[HEX]CAGCTGGGCGCCTGCCTTGACC[BHQ1] | 60 |
| rs6557168 | 5′-CAACAATTTTGGCAAGCAGTC-3′<br>5′-CACCTTCAGTTCACACAGTCTA-3′<br>[FAM]TATCTGCTGAGAAAATATAACCACATA[BHQ1]<br>[HEX]TATCTGCTGAGAAAATATAACCATATAA[BHQ1] | 61 |
| rs53576 | 5′-CGGGCACAGCATTCATGGAA-3′<br>5′-AGGAGGCCTGGTTTGAACTGT-3′<br>[FAM]TGTTTCTGTGGGACTGAGGATCC[BHQ1]<br>[HEX]TGTTTCTGTGGGACTGAGGACCC[BHQ1] | 60 |
| rs12720071 | 5′-TTAGATCTTGAAAGCACAACACAC-3′<br>5′-CAGGCAGTACCAATGCTCTTT-3′<br>[FAM]CACAAATGCAGTTTCAAAAATGAATCG[BHQ1]<br>[HEX]CACAAATGCAGTTTCAAAAATGAACCG[BHQ1] | 60 |
| rs806377 | 5′- CATGGGTAACTTCTATCATCACTAAG-3′<br>5′- GGTCCAGTAGGTGATGGCT-3′<br>[FAM]ACCAGTTCTGCACACCTTTCC[BHQ1]<br>[HEX]ACCAGTTCTGCACACCTCTCC[BHQ1] | 60 |

| | | |
|---|---|---|
| rs4522666 | 5′-CGTCCTAAGCAGCGAGCTT-3′<br>5′-GTGTGCCGTGCTTTGCATTT-3′<br>[FAM]AAACTGATTTCTTGACCCACGGT[BHQ1]<br>[HEX]AAACTGATTTCTTGACCCATGGT[BHQ1] | 65 |
